# Emotional Dysregulation in Preschool Age Predicts Later Media Use and Gaming Disorder Symptoms in Childhood

**DOI:** 10.3389/fpsyt.2021.626387

**Published:** 2021-06-17

**Authors:** Frank W. Paulus, Karen Hübler, Fabienne Mink, Eva Möhler

**Affiliations:** Department of Child and Adolescent Psychiatry, Saarland University Hospital, Homburg, Germany

**Keywords:** Emotional Dysregulation, Gaming Disorder, media use, preschool age, school age

## Abstract

**Background:** The aim of this study was to evaluate the role of early Emotional Dysregulation (ED) at preschool age as a risk factor or predictor of later media use behavior and Gaming Disorder (GD) in school age.

**Methods:** 80 patients (63.7% male; mean age = 4.2, SD = 1.23) who had attended a special outpatient program for preschoolers at measuring point time t1 were contacted at measuring point time t2 (mean age = 9.2, SD = 2.03). At t1, the comprehensive clinical assessment comprised Child Behavior Checklist—Dysregulation Profile (CBCL-DP). At t2, parents completed a questionnaire on their children's media availability, usage times, and GD.

**Results:** ED predicts a more intense use of digital media in the future. The daily average screen-use time at t2 varies significantly between the groups (148 min for children with ED at t1 and 85 min for children without ED at t1). The intensity of media use can be considered a significant predictor for the presence of a GD in dimensional assessment. When GD is classified categorically, according to the DSM-5 criteria, there is no significant correlation between ED and later GD diagnosis, neither between screen-use time and GD diagnosis. However, at dimensional level, preschool children with ED show significantly higher GD symptom scores at 9 years of age.

**Conclusion:** ED at preschool age is strongly associated with time spent video gaming and GD symptoms 5 years later. Our results strongly indicate that emotion dysregulation in preschool children is a risk factor for later problematic video game playing behavior. This strengthens the concept of ED in the etiology of media use and provides potential targets for early GD prevention.

## Introduction

Emotional Dysregulation (ED) is characterized by difficulties in understanding, accepting, and dealing with emotions [i.e., emotion regulation; (1)]. Due to these deficits, children and adolescents with ED symptoms often show little flexibility and spontaneity, with a lack of control and disruptive behavior (2). Findings suggest that ED may influence the development and course of various disorders, including different substance use disorders [e.g., alcohol, cocaine, or nicotine dependencies; (3–8)] as well as behavioral addictions [e.g., pathological gambling; (9)].

These named deficits in emotion regulation also lead to assume that some individuals try to mitigate, facilitate, or avoid (especially unpleasant, negative) emotional experiences by engaging in video games as distraction (10, 11). More specifically, it is believed that children and adolescents with ED use video game playing as a maladaptive coping strategy to deal with negative emotions (10). In addition, the disruptive and often poorly controllable behavior of children with ED can also make them less likely to socialize in preschool and school, which may lead to uncertainty in face-to-face interactions. In this respect, the lack of direct interaction with others in video games can reduce social insecurity in such situations. This probably leads to a tendency for children with low social skills and especially children with ED to prefer the internet in general but also video games in particular as a place for social interactions rather than real-life interactions (12).

With regard to early media use, some studies indicate that use of digital media might positively influence attitudes to learning and reading skills (13–18). At the same time, digital media and especially computer games are entertainment products *with clinical relevance*.

Various studies suggest that the use of digital media can also affect children negatively in their physical, social, and psychological development [(19–29); for current reviews, see (30–32)].

Especially video gaming has increased enormously in recent years (33). Gentile et al. (34) showed in a longitudinal design that more time spent gaming is a significant predictor of a later Gaming Disorder (GD). The Internet Gaming Disorder (IGD) was first recorded in the DSM-5 and is characterized by nine diagnostic criteria: excessive involvement in Internet gaming, withdrawal symptoms, tolerance development, loss of interest in previous activities, loss of control, continued gaming despite knowledge of negative consequences, deceiving significant caregivers about the gaming's extent, use of Internet gaming to end or reduce negative feelings, and endangerment or loss of an important relationship (35). Furthermore, GD has now been included in the ICD-11 (36) which includes the main diagnostic criteria listed in the DSM-5. Whereas, population-based studies estimate the representative prevalence of IGD at 2% [a mean value of representative studies from different countries: Germany, the Netherlands, Norway, Spain, UK, the USA, and one Europe-wide study; for a systematic review, see (32)], some studies conclude that the prevalence in schoolchildren is about 8–9% or more (34, 37, 38) and likewise in healthcare utilization groups.

High prevalence rates, increased usage times, dynamic development of digital technology, and the physical, social, and psychological consequences of GD pose an increasingly important public health problem in the understanding of the development and etiology of GD, including risk factors and precursors to GD. Although playing video games seems to be more and more integrated in everyday life, there is little research on the risks of the GD development in schoolchildren.

Studies have found indications that ED seems to have a significant impact on media usage and GD (12, 39–41). Hollett and Harris (40) investigated the relationship between ED and problematic video gaming using a sample of 928 adult subjects. They identified two dimensions of ED, i.e., difficulties with impulse control and limited access to emotion-regulation strategies, as significant predictors of problematic video gaming. Hormes et al. (42) assumed in undergraduate students (20 years of age) that disordered online social networking (“craving Facebook”) endorsed more ED, including non-acceptance of emotional responses, reduced emotion regulation strategies, and poor impulse control. In addition, Wichstrøm et al. (12) also found indications that symptoms of GD in 10-year-olds could be predicted by social skills and emotion regulation deficits in children at age 8, a rare research result in childhood.

Despite the increased research interest in the correlation of ED symptoms in children and the development of GD in adolescents, to our knowledge, no study has investigated whether ED symptoms in preschool age can predict GD or GD symptoms in school age. Our basic assumption is that the relation between ED and GD starts even earlier in development.

More specifically, we investigated the following hypotheses:

1. Preschool-aged children with ED meet the criteria of GD or show higher GD symptom scores in school age compared with children without ED in pre-school age.
2. Children with ED in preschool age will use media in school age longer than children without ED at preschool age. The duration of media use is a significant predictor for the presence of a GD in school age.

## Materials and Methods

### Participants

Participation in the study was voluntary and there was no financial compensation. All children and their parents gave informed consent. The local ethics committee approved the study.

**Figure 1:**
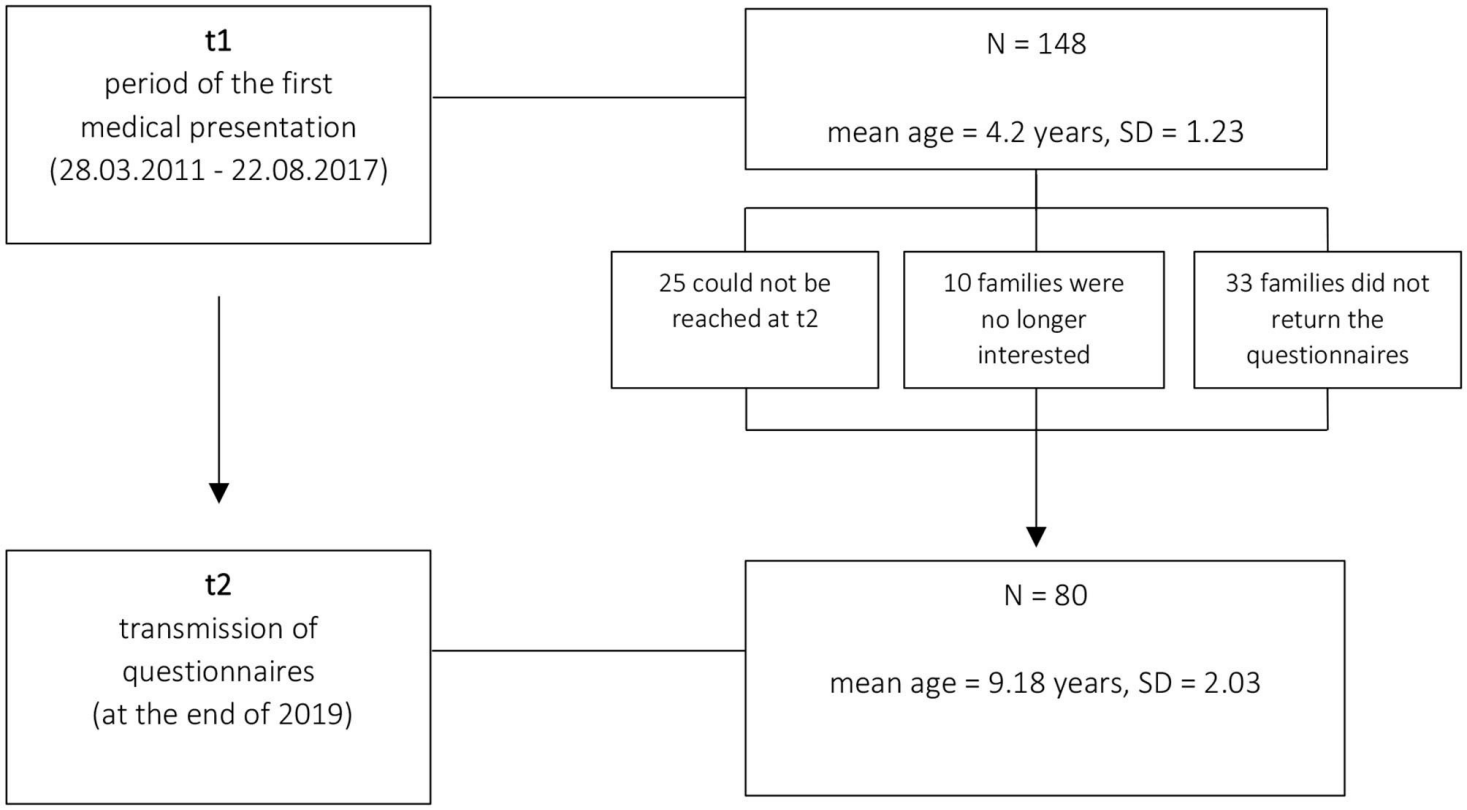
Schematic overview of the methodical approach.

In the present study, we used a quasi-experimental design with two measurement points. The study group included all young children who had attended the preschool special outpatient clinic of a Clinic for Child and Adolescent Psychiatry between 2011 and 2017, regardless of the diagnosis made (measuring point time t1). These families were contacted again at measurement time t2 (at the end of 2019) with a cover letter and a questionnaire. Of the original 148 children, 25 families could not be reached at t2. Of the remaining 123 children, 10 families expressed no interest, 113 families confirmed their participation by telephone, of which 33 did not return the documents despite repeated reminders and inquiries (for a schematic overview of the methodical approach, see Figure 1). The study was finally conducted with *N* = 80 (70% of the original children) subjects (63.7% male; mean age (t1) = 4.2, SD = 1.23, min = 1.4, max = 6.9). These 80 patients were contacted again at the end of 2018 (mean age = 9.2, SD = 2.03, min = 4.6, max = 13); there was no further personal patient presentation at measuring time t2. The average time difference t1 – t2 is therefore M = 4.9 years (SD = 1.64).

Because all the children had previously attended child and adolescent psychiatry, most participants (*N* = 76) in the study at t1 had a diagnosis with at least one disorder defined by ICD-10. The most frequent diagnosis was that of the Oppositional Defiant Disorder with *N* = 31 (ICD-10: F91.3), followed by Attention Deficit Hyperactivity Disorder with *N* = 21 (ICD-10: F90). In addition, *N* = 19 children suffer from non-organic insomnia (ICD-10: F51), *N* = 15 children suffer from specific developmental disorder of motor function (ICD-10: F82), and *N* = 13 children had a diagnosis of specific speech articulation disorder (ICD-10: F80.0). Other diagnoses that were given multiple times include the expressive language disorder with *N* = 10 (ICD-10: F80.1), the receptive language disorder with *N* = 4 (ICD-10: F80.2), feeding disorder of infancy and childhood with *N* = 6 (ICD-10: F98.2), and social anxiety disorder of childhood with *N* = 4 (ICD-10: F93.2).

In addition, IQ data were available for 71 of 80 subjects in t1 (mean IQ (t1) = 100.61, SD = 17.16). Due to the large variability in age, different test procedures were used to measure IQ [WIPPSI-III (56.3%), K-ABC-II (13.8%), SON-R 2,5–7 (8.8%), and other tests (21.2%)].

At t2, most children were attending elementary school (51%), followed by high school (19%), community schools (13%), day care (10%), special schools (6%), and Waldorf schools (1%). The majority of the children (68%) are living with both their biological parents; 24% of them are living with only their mother (and her partner) and one child is living with only its father (and his partner). Also, 5% of the children are living in foster care and 3% are living in a different living situation. *N* = 11 (13.8%) of the participants were taking medication, including Methylphenidate (3.8%), Atomoxetine (1.3%), or other medication (11.3%), including Asthma spray (1.3%), Guanfacine (1.3%), Opipramol (1.3%), Melatonin (1.3%), MTX (1.3%), Naproxen (1.3%), Dekristol (1.3%), Sulgen (1.3%), Vomex, and Salbutamol (1.3%).

### Instruments and Procedure

#### Assessment of Emotional Dysregulation

We used the Child Behavior Checklist [CBCL 1½−5; (43)] to assess ED in pre-school children at t1. CBCL 1½−5 is one of the most commonly used tools for assessing developmental psychopathology in children and adolescents (43–47). From the 99 items of CBCL 1½−5, seven scales (Emotional Reactivity; Anxiety/Depressive; Physical Complaints; Social Withdrawal; Sleep Problems; Attention Problems and Aggressive Behavior) and three superordinate scales are formed, which represent External, Internal, and Total Problem Score. Good reliability and validity have been reported for the CBCL/1.5–5; Cronbach's alpha of the superordinate scales is above 0.86 (43). We assessed deficits in emotional regulation with the Deficient Emotional Self-Regulation Profile (DESR), which is characterized by simultaneous increases (between 1 and 2 SDs) on the three syndrome scales anxiety/depression, aggression, and attention (48, 49). Furthermore, we used the Child Behavior Checklist—Dysregulation Profile (CBCL-DP) as a more severe form of the ED profile. CBCL-DP is described by simultaneous extreme values (more than 2 SD) on the three syndrome scales [(49–51), S. 192]. For categorization, the respective *T*-values were used, *T*-value >60 and <71 for DESR profile and *T*-value >70 for Dysregulation profile. In general, the DESR profile, as well as the CBCL-DP, are established diagnostic procedures for identifying ED (50–55).

#### Assessment of Media Usage Behavior

At t2, parents were sent a questionnaire assessing media use and GD. The questionnaire included items referring to media use (i.e., time spent with TV and computer or video games during the week and on weekends, availability of computer access, child's ownership of a video game console or a handheld video game) and items measuring GD. To assess media usage time, parents were asked how long their child used electronic devices such as computers, laptops, smartphones, tablets, or game consoles on average every day—separately for working days and the weekend (with the following response categories: 0, 1–30, and 30–60 min; 1–2, 2–3, 3–5 h, 5–7 h, and more than 7 h). The calculation of the media usage time in minutes was done by determining the respective category mean (0 min, 15 min, 45 min, 90 min, 150 min, 240 min, 360 min) and 450 min for “more than 7 h” at the presumption, that the mid point of each interval is used as the best and most robust estimate of the answer category.

For GD, parents should answer nine questions (e.g., “Does your child become restless, irritable, moody, angry, anxious or sad when he or she has no opportunity to play?”) on a four-level Likert scale from “never” to “always.” The questions were formulated according to the criteria proposed by Petry et al. (56), with each question comprising one of the DSM-5 criteria relating to IGD. A total score was calculated by adding up the nine items, and mean values by dividing the sum value by nine; missing items were replaced by mean values. To assess the performance of nine GD-items and the GD total score, an item analysis was performed and the reliability was determined. Reliability was high with Cronbach's α = 0.883. As confirmed by a reliability analysis, total reliability did not increase significantly by eliminating any of the nine items. In addition, discriminatory power analyses ensured a value of *r* >0.5 for every item. Thus, all nine items remained in the scale for the calculations. Since each item of the questionnaire includes one of the DSM-5 criteria regarding IGD, the DSM-5 classification criteria were applied, according to which at least five of the nine items must be fulfilled to comply with GD. An item was considered fulfilled if either “often” or “always” was selected on the four-level Likert scale (categorical value: GD yes or not). Besides this categorical assessment (GD: yes or no), a dimensional conceptualization of GD was calculated by adding up the nine items for a GD symptom score (never = 1; sometimes = 2; often = 3; always = 4 with individual values between 9 and 36). For more details on psychometrics properties of the used nine GD-items, see Table 1.

**Table 1:** Item characteristics of the nine GD criteria (*N* = 80).

**DSM-5 criteria: Gaming disorder (GD)**	**M** **(SD)**	**Item difficulty**	**Discriminatory power**	Cronbach's alpha^*^
1 Preoccupation: The child thinks about gaming when it is not playing	1.50 (0.827)	0.17	0.716	0.875
2 Withdrawal symptoms: The child is irritable, anxious, sad when devices are taken away	1.41 (0.706)	0.14	0.737	0.869
3 Tolerance: Impression of intensified media usage	1.90 (0.963)	0.30	0.627	0.895
4 Addictiveness: Child wants to play less, but does not manage	1.21 (0.520)	0.07	0.777	0.866
5 Loss of other interests: Child quits other activities	0.136 (0.767)	0.12	0.872	0.852
6 Psychosocial problems: occurrence of sleep deprivation, unpunctuality, disputes, neglect of chores	1.24 (0.621)	0.08	0.777	0.864
7 attempt to deceive: Child hides gaming from family members	1.28 (0.477)	0.09	0.630	0.877
8 Escapism: Child uses gaming to escape or relief negative mood	1.14 (0.470)	0.05	0.638	0.877
9 Impairment: Child has jeopardized or lost school performance or social relationships	1.24 (0.641)	0.08	0.864	0.855

* *The value of Cronbach's alpha of each item indicates the value of Cronbach's alpha when that particular item is taken out of the equation. Cronbach's alpha for all nine items equals α = 0.883*.

### Statistical Analysis

Data were analyzed by IBM SPSS Statistics Version 26. Categorical variables were analyzed by Fisher–Yates test (ED and media devices) and binary logistic regression (ED as predictor of a future GD diagnosis). *T*-tests were performed for group analyses of continuous variables. If the requirements for a *t*-test for independent samples were not met, a Mann–Whitney *U*-test was calculated (ED and ordinally scaled daily average media usage). For modeling the relationships within the data, univariate linear regression was conducted (ED as predictor of media usage times, media usage times as predictor of GD). A significance level of 0.05 was used for all statistical tests.

## Results

### Emotional Dysregulation

*N* = 11 children (14%) had deficits in the regulation of emotions (mean age = 8.91 years, SD = 1.49, min = 7.08, max = 11.25). Of these, *N* = 7 (9% of all participants) showed increased values of between 1 and 2 SDs on the three syndrome scales anxiety/depression, aggression, and attention of CBCL, thus fulfilling the criteria of the Deficient Emotional Self-Regulation Profile (DESR). *N* = 4 children (5%) showed simultaneous extreme values (more than 2 SD) on the three syndrome scales and thus corresponded to the CBCL-DP. The gender comparison showed that *N* = 9 boys (18%) and *N* = 2 girls (7%) had deficits in the regulation of emotions. However, this descriptively discernible difference of ED between boys and girls did not reach statistical significance (Fisher's exact test: *p* = 0.31). With regard to medication, only one child with ED was taking medication (i.e., Opipramol). All children with ED (*N* = 11) had at least one disorder defined by ICD-10. The most common diagnosis was the conduct disorder with oppositional defiant behavior (ICD-10: F91.3; *N* = 8), followed by attention deficit hyperactivity disorder (ICD-10: F90.0; *N* = 5). For more details on comorbidities of children with ED, see Tables 2, 3.

**Table 2:** Listing of ICD-10 diagnoses per subject with ED.

**Subjects with ED**	**ICD-10 diagnoses**	**Type of ED**
1	F90.1, F93.0	DESR
2	F91.3, F51.0	CBCL-DP
3	F91.3	DESR
4	F51.0, F80.0, F45.8, F93.2	CBCL-DP
5	F91.3, F90.0V, F80.0V	DESR
6	F82, F80.0, F51.0, F52.0, F91.3	DESR
7	F91.3, F90.0V	CBCL-DP
8	F93.8, F34.1V	DESR
9	F91.3, F90.0V	DESR
10	F34.1, F91.3, F90.0, F51.5, F80.0	CBCL-DP
11	F90.0, F91.3	DESR

*2 children had a total of 5 disorders, 1 child had a total of 4 disorders, 1 child had a total of 3 disorders, 6 children had a total of 2 disorders, and 1 child had 1 disorder in t1*.

**Table 3:** Frequency of individual ICD-10 diagnosis in children with ED.

**Disorders in children with ED in t1 (diagnosed with ICD-10)**	**Frequency**
F91.3	8
F90.0	5
F80.0	4
F51.0	3
F34.1	2
F90.1	1
F93.0	1
F45.8	1
F93.2	1
F82.0	1
F52.0	1
F93.8	1
F51.5	1

### Access to Media Devices and Usage Time

**Figure 2:**
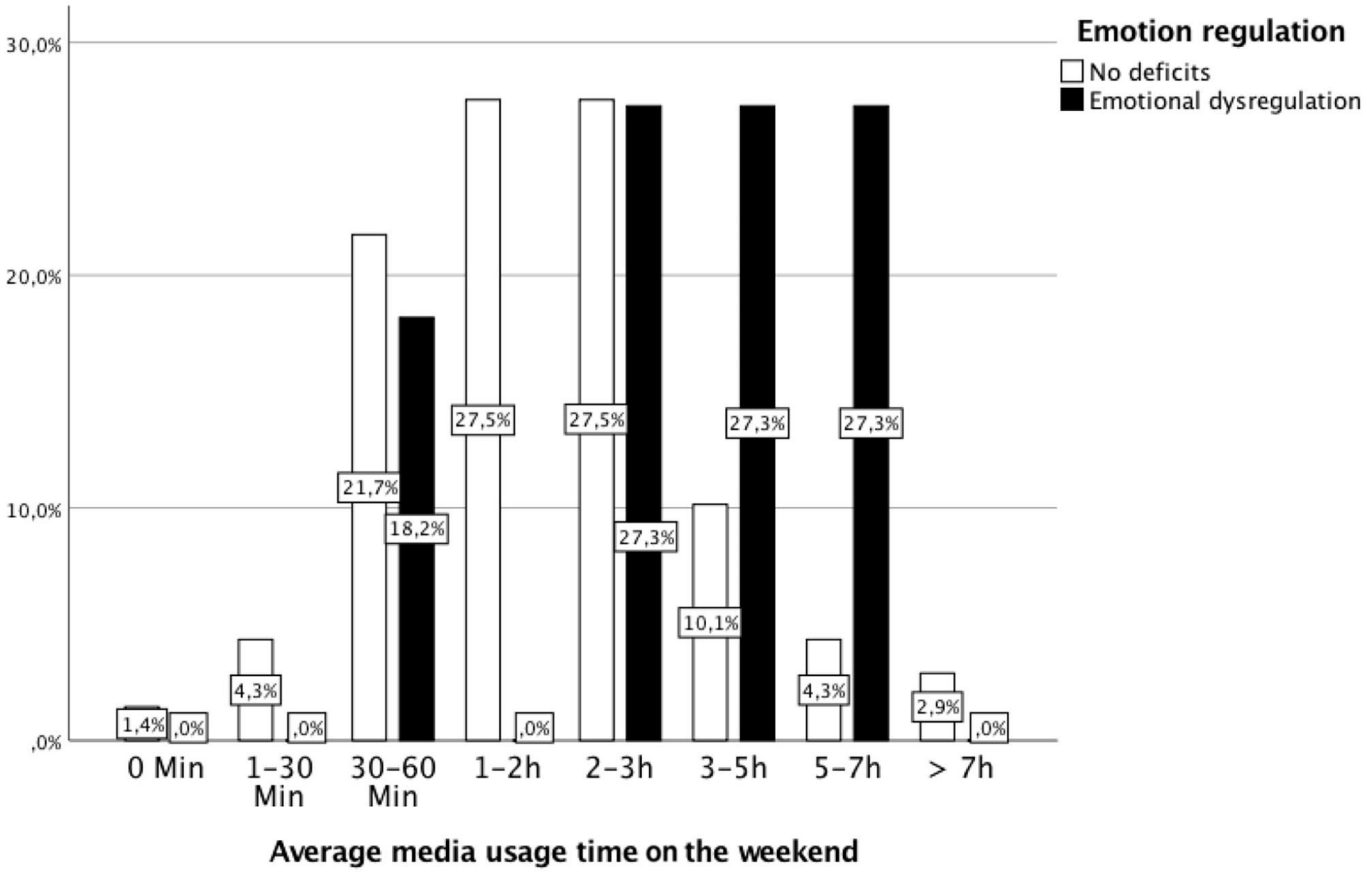
Daily average media usage time at the weekend by children with ED and without ED. Children with ED used media significantly longer at weekends than children without ED (Mann–Whitney *U*-test: M_rank_ with ED = 55.50, M_rank_ without ED = 38.11; U = 214.5, Z = −2.36, *p* < 0.018).

**Figure 3:**
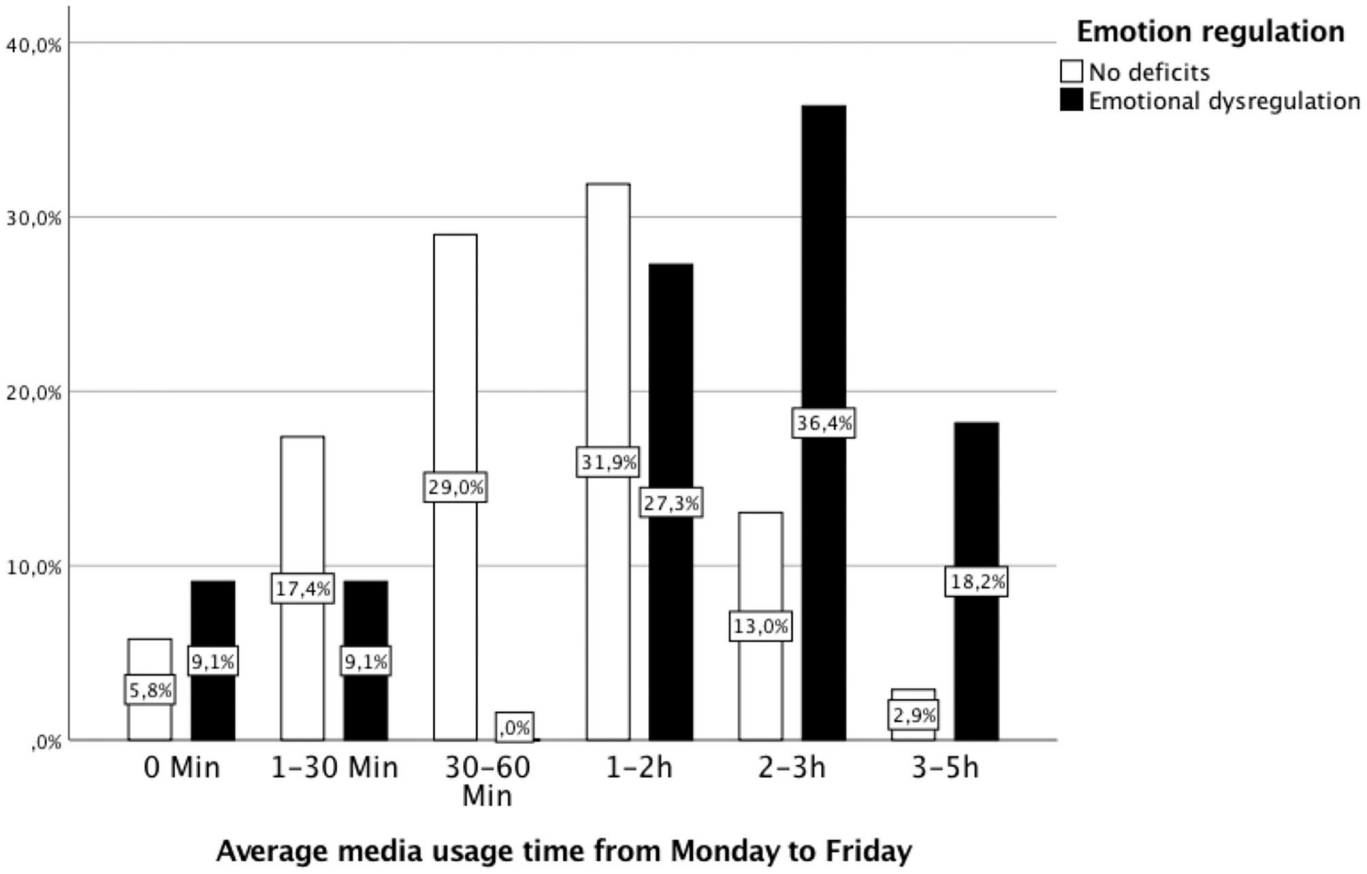
Daily average media usage time from Monday to Friday by children with ED and without ED. Children with ED used media significantly longer on weekdays than children without ED (Mann–Whitney *U*-test: M_rank_ with ED = 55.77, M_rank_ without ED = 38.07; U = 211.5, Z = −2.41, *p* = 0.016).

In terms of access to a computer/laptop (*p* = 0.974), smartphone (*p* = 0.933), Smartwatch (*p* = 0.362), tablet (*p* = 0.751), stationary game console (*p* = 0.169), portable game console (*p* = 0.726), and television (*p* = 0.100), there were no significant differences between children with and without ED (for more details, see Table 4). As shown in Figures 2, 3, children with deficient emotional regulation used media significantly longer on weekdays (*U* = 211.5, *Z* = − 2,413, *p* < 0.05) as well as on weekends (*U* = 214.5, *Z* = − 2.36, *p* < 0.05) than children without ED, confirming the second hypothesis. The calculation of media usage time at t2 presented children with at t1 diagnosed ED to have elevated usage times compared with those cases, when an ED was not determined. On weekdays, children with ED used media for averagely 124 min per day (vs. 68 min for children without ED, *p* = 0.003). On the weekend, media was used more frequently by children with ED, averaging 209 min per day (vs. 129 min for children without ED, *p* = 0.019). For details, see Table 5.

**Table 4:** Access to different media devices in children with ED and children without ED.

**Media devices**	**Children with ED (*n* = 11)**	**Children without ED (*n* = 69)**	**Statistics (*Fisher's exact test*)**
Computer/laptop	*n* = 5 (45.45%)	*n* = 31 (44.93%)	*p* = 0.974
Smartphone	*n* = 7 (63.64%)	*n* = 43 (62.32%)	*p* = 0.933
Smartwatch	*n* = 1 (9.09%)	*n* = 2 (2.90%)	*p* = 0.362
Tablet	*n* = 7 (63.64%)	*n* = 39 (56.52%)	*p* = 0.751
Stationary game console	*n* = 6 (54.55%)	*n* = 21 (30.43%)	*p* = 0.169
Portable game console	*n* = 4 (36.36%)	*n* = 20 (28.99%)	*p* = 0.726
Television	*n* = 11 (100%)	*n* = 64 (92.75%)	*p* = 0.100

**Table 5:** Differences in media usage time in minutes in children with ED and without ED.

**Period of usage time**	**Children with ED** **(*n* = 11)**	**Children without ED** **(*n* = 69)**	**Statistics (*t*-test)**
Weekdays (Monday–Friday)	M = 124 min (SD = 77.68)	M = 68 min (SD = 52.76)	t(78) = −3.075; *p* = 0.003; d = 0.851
Weekends (Saturday and Sunday)	M = 209 min (SD = 122.91)	M = 129 min (SD = 98.27)	t(78) = −2.421; *p* = 0.018; d = 0.719
Daily average^*^	M = 148 min (SD = 88.31)	M = 85 min (SD = 62.06)	t(78) = −2.949; *p* = 0.004; d = 0.828

* *Daily average usage time was calculated by adding up five times the usage time on weekdays and two times the usage times on weekends divided by seven*.

Based on the finding that longer usage times (34) as well as ED (39, 40) are significant predictors of GD, we investigated exploratively whether ED at t1 is also predictive for longer media usage times at t2. A linear regression with dimensional conceptualization of ED as predictor and the metric scaled variable of the subjects' duration of media use during the daily average as criterion showed that preschool ED at t1 significantly predicts usage times of digital media use per week (*F*_(1)_ = 8.698, *p* = 0.004, *R*^2^ = 0.100, *R*^2^_adjusted_ = 0.089) 5 years later at t2. To test our second hypothesis, that duration of media use is a predictor of a GD, further linear regression analyses were carried out, which showed that the time criterion of media usage behavior is a significant predictor for the presence of a GD dimensionally (*F*_(1, 78)_ = 22.863, *p* = 0.000, *R*^2^ = 0.227, *R*^2^_adjusted_ = 0.217). Categorically, it did not explain a significant amount of the variance in GD (*F*_(1, 78)_ = 2.679, *p* = 0.106, *R*^2^ = 0.033, *R*^2^_adjusted_ = 0.021).

### Media Use Behavior

If gaming behavior is assessed dimensionally and the mean values of the two groups are compared, a descriptive difference between children with ED (M = 15.82, SD = 4.08) and children without ED (M = 11.71, SD = 5.05) is already apparent. This difference is also statistically significant (*t* = −3,0; *p* =0.004; *d* = 0.895). However, if gaming behavior is categorically classified according to the DSM-5 criteria for GD (diagnosis when >4 criteria from 9), only one of the 11 children with ED (9.09%) and three of the 69 children without ED (4.35%) met the criteria for a diagnosis of GD. A binary logistic regression showed no significant correlation between ED and future GD diagnosis (Wald(1) = 31.989*, p* = 0.51; n.s.). The differences between children with ED and children without ED on dimensional and categorical assessment of gaming disorder are listed in Table 6.

**Table 6:** Differences between children with emotional dysregulation and children without emotional dysregulation on dimensional and categorical assessment of gaming disorder.

**Assessment of GD**	**Children with ED** **(*n* = 11)**	**Children without ED** **(*n* = 69)**	**Statistics (*t*-test)**
Dimensional GD	M = 15.82 (SD = 4.08)	M = 11.71 (SD = 5.05)	t(68) = −3,0; *p* =0.004; *d* = 0.895
Categorical GD	Children with GD *n* = 1 (9.09%)	Children with GD *n* = 3 (4.35%)	Wald(1) = 31.989 *p* = 0.51; n.s.
	Children without GD *n* = 10 (90.90%)	Children without GD *n* = 66 (95.65%)	

*Dimensional GD was calculated by adding up the response characteristics of 9 GD symptoms to the GD symptom score (1 = never, 2 = sometimes, 3 = often, 4 always). A categorical GD was diagnosed only if at least 5 out of 9 items were answered with “often” or “always.”*

## Discussion

In recent years, the everyday use of digital media and especially the increase in playing video games seems to have a huge, and potentially negative, impact on child and adolescent development, to the point of a manifest GD (35, 36). A potential factor, which probably affects the development of GD, is ED (12, 40). All studies carried out on this subject so far investigated the influence of ED in schoolchildren, adolescents, or young adults on the development of GD. To the best of our knowledge, this is the first study to examine the impact of preschool ED on media use and GD in later childhood. To potentially find early prevention possibilities, we conducted the study with the aim of examining the predictive value of ED in preschool age for the development of GD in school age.

In summary, the results show higher GD symptom scores (dimensionally) in school age for children with preschool ED compared with children without ED in preschool age. ED does not predict a diagnosis of GD (categorically) (hypothesis 1). Children with ED at preschool age have significantly longer media use times 5 years later, than preschool children without emotion regulation difficulties. Temporal excessive video game playing behavior at school age is correlated with higher GD symptom scores (dimensionally), but not with the presence or absence of a GD diagnosis (categorically) (hypothesis 2).

It amounts to a difference between a dimensional and a categorical approach to GD. ED in preschool age, as well as screen time use predict higher GD symptom scores on a dimensional scale, but do not predict a GD diagnosis on a categorical scale. Possible explanations for these differences could lie in (a) larger required samples to yield sufficient statistical power. (b) It may be speculated that the GD criteria of the DSM-5 do not apply as well to the according age group (9 years) compared with adolescents or young adults (developmental adequacy). The item analysis of the nine GD criteria supports this speculation: the means lie in the lower range (see Table 1). The item difficulties are all with one exception under 0.20. (c) A reflection of the differences between the dimensional and the categorical approach of diagnostic and psychopathology is fundamental. A dimensional approach allows the clinician more latitude in assessing the severity of a condition and does not imply a concrete threshold between “normality” and the disorder, such as GD (57–59).

The reported increase of usage time in children with ED can be attributed to the ED profile because in general, children with psychiatric disorders use digital media significantly longer (60). Children with ED may use video game playing as a maladaptive coping strategy to deal with negative emotions. Because ED is characterized by difficulties in understanding and accepting unpleasant emotions, it is assumed that children with ED often try to avoid such emotional experiences, e.g., by using excessive video gaming to escape psychological difficulties (10, 11, 40, 42). Therefore, young children with ED should be seen as being at risk of developing a GD. This risk may be further increased by the game-associated induction of positive feelings. During the game, children and adolescents feel particularly free and heroic, which in turn leads to a stabilization of their self-esteem. They have a direct sense of achievement and self-efficacy, make social contacts more easily than in real life, and are able to escape the emotional difficulties of the real world, whereas the latter may be a maladaptive coping strategy leading to more excessive gaming (10). Considering all these aspects, in regard to future prevention, it could be a promising approach to limit the time spent on video games. More specifically, children prone to difficulties in dealing with negative emotions should be restricted or monitored more closely in their video game playing behavior. This falls in line with the proposition of Donald et al. (61) to reduce video gaming by restricting access to devices (despite them having considered ED as a result of GD instead of ED predicting GD, as we have found). At the same time, more adequate coping and action alternatives should be offered that both act as an adaptive strategy for dealing with negative emotions and can contribute to experiencing positive feelings.

Furthermore, in the context of the assessment of our second hypothesis, the analyses show that the time criterion of media usage behavior in school age is associated with the existence of a GD, in dimensional GD conceptualization. Therefore, our second hypothesis and the findings from Gentile et al. (34) could be confirmed, whereby the time criterion could be assumed as a manifest risk factor for the development of a GD. We conclude that the time aspect of video game playing behavior should be considered as playing a more prominent role in the development of GD. The tolerance development in DSM-5 is time associated (e.g., feeling the need to play for increasing amounts of time, augmentation of play time). This assumption is strengthened by various findings of other authors, which show a significant association between the usage time of video games and GD (62–66). Again, the time limitation of video game usage behavior, as well as the usage of alternative and adaptive activities, could be useful for the prevention of GD. To our knowledge, there are no efficient studies of possible prevention measures for GD in the context of ED. Therefore, it would be of great interest to undertake further research on prevention measures, e.g., time limitation. Studies indicate that daily use of digital media is not limited to school age and adolescence but also widespread in pre-school age, e.g., Vandewater et al. (67) reported 16% of 5–6-year-old pre-school children playing video games daily. Mendoza et al. (68) reported that already 2–5-year-old children used a computer daily. Thus, effectiveness studies on prevention programs for GD in the context of ED would also be highly relevant for children of younger age. To explore the relation between ED and GD (hypothesis 1), we used a dimensional (i.e., symptom score of GD), as well as a categorical (i.e., diagnosis of a GD), assessment of this construct. In the dimensional assessment of video game playing behavior, children with ED in preschool age show a higher GD symptom score at school age than those without ED. These findings largely confirm our hypothesis 1, as do the findings of Hollett and Harris (40) and Wichstrøm et al. (12). These findings suggests that early detection and treatment of ED could have a preventive effect on the development of GD. Especially in young age, increasing social support with a family- and parent-based approach could reduce Internet addiction (41). Nevertheless, to make more detailed statements in this regard, further research is needed.

## Limitations

One restriction of the present study is the limitation to parental reports. Parents may report their children spending less time using media, either because of underestimation or because of omitting time spent on media outside home, e.g., with friends. They may also react biased toward social desirability, being aware that their children should not spend that much time online. Furthermore, for future research it would be interesting to consider parents' media use as a mediator variable.

Another limitation of this study is that we only assessed the total usage time of modern electronic media, without differentiating for computer/laptop, smartphone, smartwatch, tablet, gaming consoles, and television. It was also not differentiated between pedagogically valuable content and problematic content. For the calculation of the media usage time in minutes based on the assessed answer categories, we performed a transformation of each category in an average value in minutes with the presumption, that the mid point of the interval serves as the best estimate of the answer category.

Our study does not provide any data on socioeconomic status (SES) and psychiatric family history; therefore, an influence of SES and psychiatric family history can neither be demonstrated nor excluded. Patients of our preschool program are living in the surrounding regions which are characterized by a quite homogenous socioeconomic status. Future studies considering SES and psychiatric family history as an important variable are underway.

In addition, the group of children actually meeting criteria for ED was small. Retention rate from the original sample was 70% only; however, a time span of 5 years was covered. Also limiting this study in its generalizability is the fact that we investigated a clinical sample. Finally, our study uses a quasi-experimental design. It would be of great value to conduct an a priori defined experimental long-term study with a larger sample, in which the course of possible development of GD in the context of ED would be recorded. In addition, the gender comparison in children with ED did not reach statistical significance and medication use was also unevenly distributed across the sample, which is probably due to the small size of the sample. Therefore, a larger sample would possibly also allow the examination of these variables as covariates in statistical analyses, which would allow the results of the present study to be illuminated against the background of further potential influencing variables.

## Conclusion

Our results suggest that preschool ED symptoms and duration of media use predict a higher score of GD symptoms in schoolchildren. Therefore, identifying ED could be the first step for parents to reduce the likelihood for the emergence of GD in schoolchildren. For example, parents should strive to convey the value of self-control and offer training in self-reflection with the aim of promoting self-regulated behavior. Therefore, one noteworthy strength of the present study is that it explores the rarely investigated and relevant area of risk factors of children's video gaming behavior. Excessive computer use and GD becomes a dysfunctional solution or an inadequate coping for pre-existing ED. Therefore, preventing ED contributes to the prevention of GD.

Regardless of the significant associations between ED in pre-school and later media use and GD in childhood, the question of the relationship between ED and ICD-10 nosology arises, which has not been clarified as yet. Would ED be the “Grand Unifying Theory” of psychological symptoms and disorders?

## Data Availability Statement

The raw data supporting the conclusions of this article will be made available by the authors, without undue reservation.

## Ethics Statement

The studies involving human participants were reviewed and approved by Ethikkommission der Ärztekammer des Saarlandes Ha 147/19. Written informed consent to participate in this study was provided by the participants' legal guardian/next of kin.

## Author Contributions

FP: conceptualization, implementation, data collection, statistical analysis, text creation, and discussion. KH: data collection, data entry, and ethics application. FM: statistical analyses and text creation. EM: discussion and correction. All authors contributed to the article and approved the submitted version.

## Conflict of Interest

The authors declare that the research was conducted in the absence of any commercial or financial relationships that could be construed as a potential conflict of interest.
